# Biomimetic Approaches to “Transparent” Photovoltaics: Current and Future Applications

**DOI:** 10.3390/molecules28010180

**Published:** 2022-12-25

**Authors:** Michele Pompilio, Ioannis Ierides, Franco Cacialli

**Affiliations:** Department of Physics and Astronomy and London Centre for Nanotechnology, University College London, London WC1E 6BT, UK

**Keywords:** transparent photovoltaics, semi-transparent photovoltaics, biomimetic

## Abstract

There has been a surge in the interest for (semi)transparent photovoltaics (sTPVs) in recent years, since the more traditional, opaque, devices are not ideally suited for a variety of innovative applications spanning from smart and self-powered windows for buildings to those for vehicle integration. Additional requirements for these photovoltaic applications are a high conversion efficiency (despite the necessary compromise to achieve a degree of transparency) and an aesthetically pleasing design. One potential realm to explore in the attempt to meet such challenges is the biological world, where evolution has led to highly efficient and fascinating light-management structures. In this mini-review, we explore some of the biomimetic approaches that can be used to improve both transparent and semi-transparent photovoltaic cells, such as moth-eye inspired structures for improved performance and stability or tunable, coloured, and semi-transparent devices inspired by beetles’ cuticles. Lastly, we briefly discuss possible future developments for bio-inspired and potentially bio-compatible sTPVs.

## 1. Introduction

Finding suitable renewable energy sources is one of the key challenges faced by humanity, mainly because of the impelling need to reduce green-house gases (GHGs) emissions and, thus, minimise climate change, even before the finite nature of fossil fuels becomes an issue [1].

Among renewables, solar energy is probably the one with the highest potential, based on consideration of the total radiative power reaching our planet. More solar energy hits Earth in one hour than the entire annual energy consumption on the planet in 2010 (4.3 × 10^20^ J vs. 4.1 × 10^20^ J) [2]. Over a whole year, the solar energy received on Earth is ten times larger than all of the known reserves of energy coming from oil, coal, natural gases, and uranium combined [2].

Even though the amount of energy potentially harvestable is so incredibly vast, many challenges still remain for the large-scale exploitation of solar energy as a substitute to fossil fuels, resulting in only 3.68% of the total electricity produced coming from solar power in 2021 (Source: https://ourworldindata.org/renewable-energy (accessed on 17 December 2022)).

Some of these challenges are hard to address. One of these relates, for example, to the sizeable portion of solar energy being absorbed or reflected by the Earth’s atmosphere. In principle, this problem can be solved by extra-terrestrial solar power generation, which, however, still requires significant development, both in terms of subsequent transfer to Earth installations, and of orbital positioning, maintenance, and repair costs.

Other challenges are easier to address, as they are related to the specific current development of the relevant technology, such as limitations in energy storage and distribution [3,4]. A possible solution to challenges of this sort could be found in increasing the amount of PV energy produced directly where it is needed by utilising building-integrated photovoltaics (BIPVs) or building-applied photovoltaics (BAPVs), because buildings account for around 40% of the world’s energy consumption [5].

The first category (BIPVs) describes PV devices used to substitute traditional building materials during the construction of the building itself (usually for its façade), while the second (BAPVs) describes devices added to the building after its construction (usually on its rooftop) [6]. BIPVs, using a variety of technologies (including those based on dye-sensitised cells, DSSCs [7], as well as organic [8] and/or perovskite photovoltaics [9,10]), have already found successful demonstrations. A recent demonstration is the building resulting from the collaboration between Skanska and the PV manufacturer Saule technologies (Source: https://sauletech.com/saule-technologies-and-skanska-change-construction-industry/ (accessed on 17 December 2022)).

Exploitation of the lateral surfaces of buildings for solar energy harvesting, for example, by introducing windows with PV capabilities, could increase the area of usable surfaces for this purpose, consequently leading to more than doubling the amount of solar energy produced by buildings [6]. This, however, would require a different approach to PV devices.

Traditional, opaque, PVs can, in fact, be unsuitable for many building-related applications, as is the case for implementation in windows: for these purposes, transparent and/or semi-transparent PVs are the required solution.

Semi-transparent photovoltaics can, in principle, also be of interest in so-called “agrivoltaics” applications, where solar panels are distributed in agricultural settings either in the open fields or integrated in the greenhouse architecture. In either case, careful consideration of cost/performance factors is required to ensure that the sacrifice of the cells’ efficiency, imposed by its (semi)transparency, is justified in terms of agricultural yields.

TPVs can also be used outside of large-scale energy harvesting, as in the field of vehicle-integrated PVs (VIPVs), where transparent solar panels built into the roofs, windscreens or windows of electric vehicles can improve their range or feed some of their less power-intensive equipment [11].

Last, but not least, nanostructured, biomimetic surfaces of windows can act as antireflective coatings and, thus, reduce both “light-pollution” effects on the surroundings, and even thermal loading on front-facing buildings [12].

## 2. Key Challenges

TPVs and sTPVs have different requirements to those of traditional PV devices. As their names suggest, transparency in the visible range of the electromagnetic spectrum is a key aspect for these devices. This is quantified by their (spectrally) averaged visible transparency (AVT). However, it is necessary to seek a compromise between transparency and absorption (necessary to generate excitons or free carriers and eventually a current), and even those cells that are indicated as TPVs are, in fact, partially absorbing in the visible.

There is no clear consensus on the threshold in transparency separating TPVs and sTPVs, with certain authors categorising devices with an AVT of 60% as semi-transparent [13] and those with an AVT of 65% as transparent [14]: in this review, we, therefore, consider an AVT of 60% to be the border separating the two categories.

Furthermore, achieving high AVT inevitably decreases the device’s power conversion efficiency (PCE), so a compromise between the two should be found: for example, smart windows usually require an AVT of around 50% and a PCE between 2 and 5%, whereas it is generally accepted that mobile displays need an AVT of around 80% and a minimum PCE of 5–10% [6]. In light of this, a new figure of merit has been proposed by Traverse et al. for evaluating the performance of this kind of PV cells: the light utilisation efficiency (LUE), given by the product PCE × AVT [6].

A promising way of achieving high LUE is by developing wavelength-selective solar cells, which selectively absorb in the near infrared (NIR) or ultraviolet (UV) region of the solar spectrum (or both) while transmitting visible light. It has been shown that the theoretical Shockley–Queisser limit for the efficiency of a single junction TPV device selectively harvesting radiation at wavelengths greater than 670 nm (mostly NIR) and shorter than 435 nm (mostly UV) with an AVT of 100% is 20.6% [15]. In comparison, the PCE of a non-wavelength-selective device approaches 0% for AVT close to 100% [6]. The most promising type of wavelength-selective (s)TPVs is considered to be that of organic solar cells (OSCs), both because of significant advances in the PCE achievable in opaque OSCs (up to ~19% in recent works [16,17,18]) and because of the high versatility afforded by the possibility of designing organic semiconductors absorbing in a specific range [19,20,21]: thanks to these characteristics, an impressive LUE of around 5% can be achieved in this type of solar cell [20,22]. The state-of-the-art value, as of December 2022, is 5.35% [19]. It is worth pointing out that the absorption features of perovskite semiconductors can also be tuned, thus making perovskite-based solar cells another promising candidate for further development in (s)TPVs [23].

For BIPVs, there is also the requirement of the devices being aesthetically pleasant, especially in residential or urban contexts, which is not a concern for most PVs utilised in solar farms.

**Figure 1:**
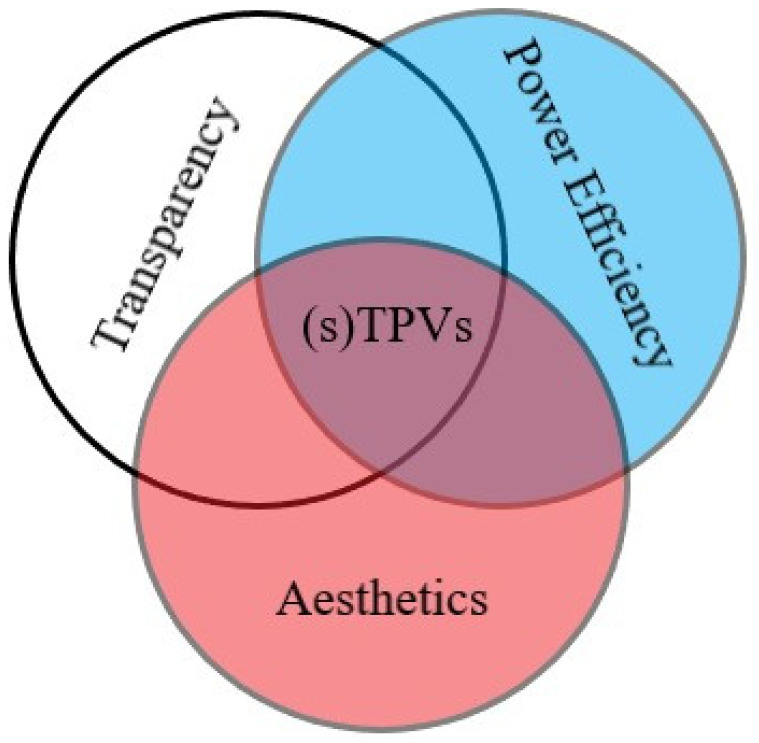
Visual schematisation of the three key factors in the design of TPVs and sTPVs.

These three key factors (AVT, PCE, and aesthetics) are the most important ones to consider when developing transparent or semi-transparent photovoltaics (Figure 1).

**Figure 2:**
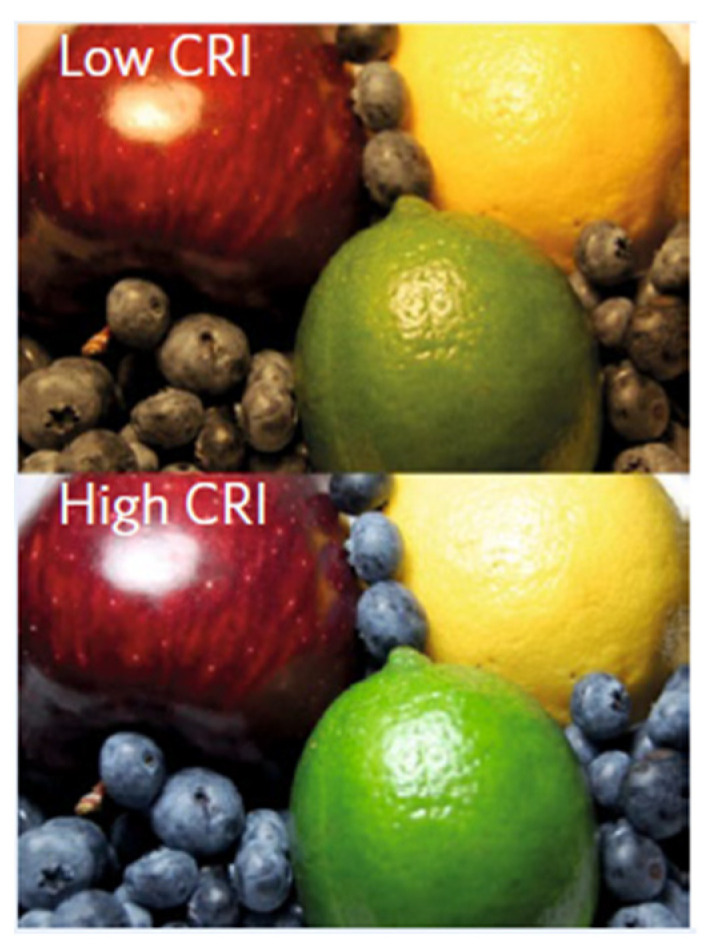
Difference in colour-rendering capabilities between high and low CRI. Reprinted with permission from Ref. [6]. 2017, Springer Nature: Nature Energy.

Another key characteristic relevant to TPVs and sTPVs is their ability to accurately transmit colours, especially for windows and vehicle applications. This is measured by their colour rendering index (CRI), which quantifies the accuracy with which an object’s colour is rendered from a light source or through a transparent medium, compared to a reference light source (e.g., a black body at a certain colour temperature). For window applications, the AM1.5G spectrum is considered to be the best standard to use [6]. CRI can assume values from 0 to 100, with higher values representing more accurate colour-rendering capabilities (Figure 2): a light source with a CRI of 70–90 is considered to be of good quality [15] and the same is true for TPVs or sTPVs.

Furthermore, as is the case for every type of PV expected to operate outside of a controlled environment, stability and resistance to deteriorating factors, such as rain, humidity, and bacteria, are also required in order to obtain high quality devices [24].

One might hope that many of these challenges might be addressed by looking at the natural world, where animals and plants have evolved a plethora of solutions to light-management and stability problems over millions of years of evolution, ranging from structural colour, aimed at camouflage or at attracting a mate, to photosynthesis-enhancing structures, to super-hydrophobic leaves [24,25,26,27]. Some of these problems, of course, also find other types of solutions in the natural world. Colouration, for example, is often obtained through pigments instead of structural colour: in plants, most of the colours come from three classes of pigments (porphyrins, carotenoids, and flavonoids) [28], while in reef-building corals the bright colouration is given by green fluorescent protein-like molecules [29]. Bio-inspired photonic structures, however, have the advantage of owing their properties more to the structure itself than to the specific materials of which they are made, making them very versatile in their implementation. For this reason, we focus on a more structural approach to biomimetic TPVs and sTPVs.

## 3. Current State

Including biomimetic or biological elements in the design of TPVs and sTPVs can be a way of finding solutions to the many challenges that are typical of such devices: such challenges relate to either, or both, the aesthetical and performance aspects (including stability), as the two are often closely related (a device with an AVT of 80% or more would have the same impact on the façade of a building as regular transparent glass, for example [6]).

In this section, we present the latest research involving biomimetic solutions that have enabled improvements to TPV and sTPV devices.

### 3.1. Optical Performance and Aesthetics

In general, (s)TPVs intended for building-integrated applications are expected to meet the additional requirement of not detracting from the aesthetics of the building on which they are placed. How this issue is handled, however, depends on the specific application in question.

In warmer regions of the world, the highest demand for electricity is registered in correspondence to higher cooling demand within buildings [13]: in such a scenario, switching from highly transparent conventional glass to sTPVs with lower AVT would decrease the amount of cooling required to keep the internal space at a comfortable temperature, which, in turn, could lead to a saving of energy of up to 12%, as shown by Li et al. in a study based on field measurements obtained in Hong Kong during the month of July 2007 [30], as well as generating energy.

The minimum AVT required for windows is usually accepted to be at 20–30% [23], so sTPVs within this range would be ideal candidates for these applications.

**Figure 3:**
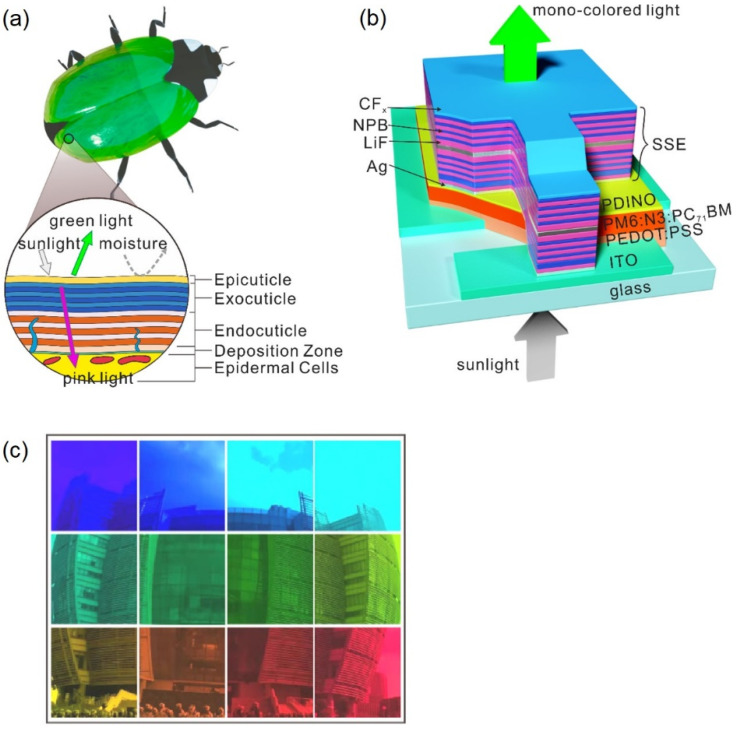
Beetles’ cuticle inspired sTPVs. (**a**) Schematic of a beetle’s cuticle. (**b**) Schematic of the spectrally selective electrodes. (**c**) Montage showing spectrally selective electrodes of different colours, obtained by tuning the thickness and number of different dielectric layers for the transmission of a specific colour. Here, PDINO stands for perylene diimide functionalized with amino N-oxide, ITO stands for indium tin oxide, PM6 stands for poly[[4,8-bis[5-(2-ethylhexyl)-4-fluoro-2-thienyl]benzo[1,2-b:4,5-b′]dithiophene-2,6-diyl]-2,5-thiophenediyl [5,7-bis(2-ethylhexyl)-4,8-dioxo-4H,8H-benzo[1,2-c:4,5-c′]dithiophene-1,3-diyl]-2,5-thiophenediyl], N3 stands for azide, PC_71_BM stands for [6,6]-Phenyl-C71-butyric acid methyl ester, and PEDOT:PSS stands for poly(3,4-ethylenedioxythiophene) polystyrene sulfonate. Adapted with permission from Ref. [31]. 2020, American Chemical Society.

Examples of semi-transparent devices with low AVT and good aesthetics have been developed by Wang et al. by reproducing light-management strategies from beetles’ cuticles in the fabrication of spectrally selective electrodes (SSEs) [31]. Some beetles exhibit bright structural colour by stacking layers of materials with different refractive indices in the same way as a Bragg mirror [27] (Figure 3a). More specifically, by combining stacks composed of several layers of LiF and N,N′-bis(naphthalen-1-yl)-N,N′-bis(phenyl)benzidine (NPB) with metal films, an anti-reflecting coating, and a hydrophobic external layer for improved stability (Figure 3b), devices with a PCE of 15.07% and peak transmittance of ~30% were fabricated (with a corresponding LUE of around 4.52%).

Furthermore, by modifying the thickness and the number of dielectric layers, it is possible to tune the colour of the resulting devices with a colour purity of almost 100% (Figure 3c). This allows for BIPVs that may require a surface of a specific colour to be used in buildings so that the intended aesthetically pleasing effects can be created without limitations.

**Figure 4:**
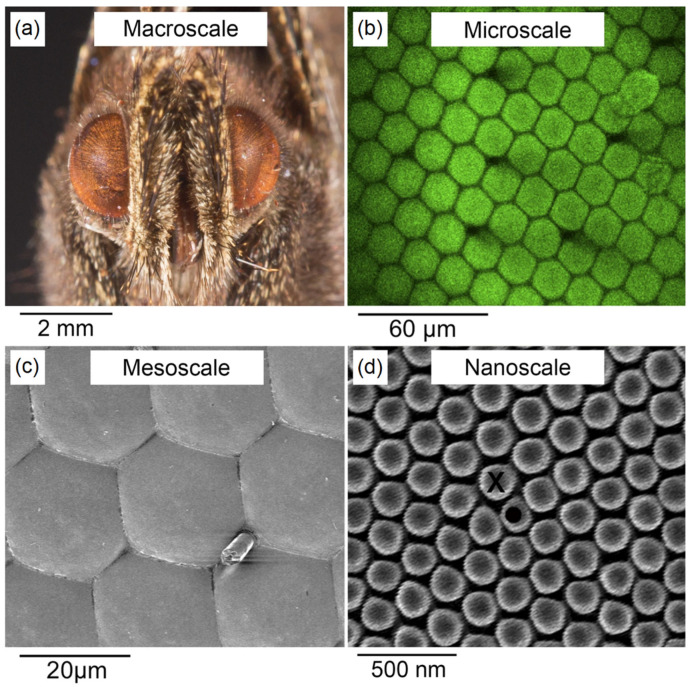
Structure of the eye of a Mourning Cloak butterfly (Nymphalis Antiopa) on different length scales. (**a**) Macroscale. (**b**) Microscale. The hexagonal close-packed pattern is clearly visible. (**c**) Mesoscale. Each hexagon of the pattern is around 20 µm wide. (**d**) Nanoscale. The average nipple diameter is 170 nm. Adapted with permission from Ref. [34]. 2016, license CC BY 4.0 (https://creativecommons.org/licenses/by/4.0/ (accessed on 19 April 2022)).

Another biological structure that has been successfully replicated in the field of bio-inspired PVs is the moth-eye. The corneas of many species of butterflies and moths exhibit nanostructures consisting of millions of nanometric-sized bumps of chitinous material with height and diameter varying from tens to a few hundred nm (Figure 4), which act as a layer with gradually changing refractive index [32]. It has been shown by using an optical multilayer model that an array of nipples with a paraboloid shape, 250 nm high, and closely packed in hexagonal domains almost completely negates the reflection of normally incident light [33]. This structure provides the insect’s eyes with anti-reflection capabilities, improving the animal’s camouflage and night vision and reducing eye wettability [32,34]. By mimicking such structures, it is possible to improve the PCE of PV devices, while also reducing light reflection, which, in turn, also lowers the light pollution impact for BIPV applications, as shown by the work of Zheng et al. [12].

In this study, they integrated a moth-eye biomimetic nanostructure into an organic semi-transparent solar cell, which resulted in a relative decrease of reflectance of 28% (from 14.3% for a flat device to 10.3%) and an increase of PCE from 3% to 3.4%, with an AVT of 46.1% (LUE = 1.57%).

**Figure 5:**
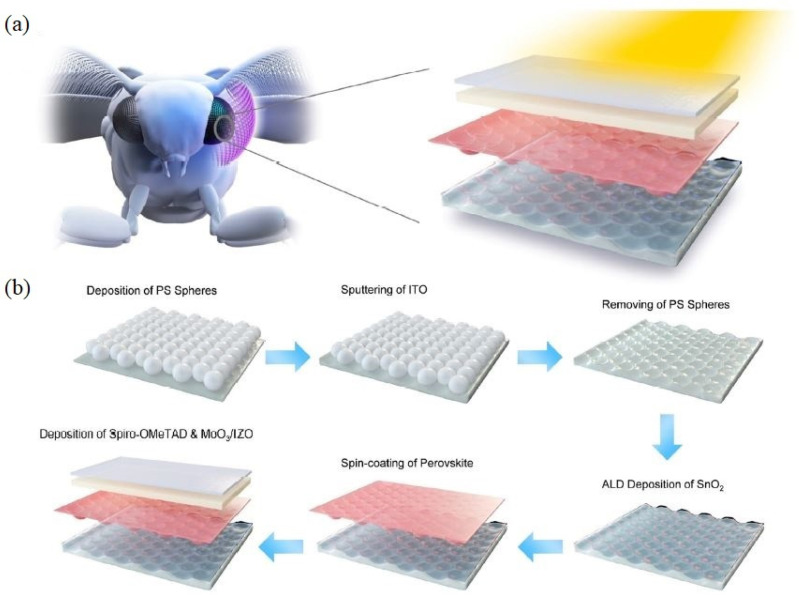
(**a**) Moth compound eye and schematic of the moth-eye inspired structure. (**b**) Illustration of the fabrication process of the sTPV device based on Cs_0.05_FA_0.83_MA_0.12_PbBr_0.33_I_0.27_ perovskite. Here, PS stands for polystyrene, ALD for atomic layer deposition, Spiro-OMeTAD for 2,2′,7,7′-Tetrakis[N,N-di(4-methoxyphenyl)amino]-9,9′-spirobifluorene, and IZO for indium zinc oxide. Adapted with permission from Ref. [35]. 2021, license CC BY 4.0 (https://creativecommons.org/licenses/by/4.0/ (accessed on 19 April 2022)).

Another successful implementation of the moth-eye nanostructure in the development of an sTPV device was presented by Zhu et al., who applied it to a Cs_0.05_FA_0.83_MA_0.12_PbBr_0.33_I_0.27_ perovskite solar cell (where FA and MA stand for formamidinium and methylammonium, respectively) [35]. In this work, the authors developed a moth-eye inspired structure with light-trapping capabilities and reduced reflectance in the spectral region where the human eye is more sensitive (Figure 5).

By utilising this structure, a PCE of 10.53% at an AVT of 32.5%, was achieved: this yielded a LUE of 3.42%, the state-of-the-art for perovskite sTPVs, as of May, 2021. For comparison, the best non-transparent perovskite-based solar cells afford a PCE well over 20% under AM1.5G illumination (Source: https://www.nrel.gov/pv/cell-efficiency.html (accessed on 17 December 2022)).

In comparison, organic-based sTPVs can reach a PCE as high as 10.8% with an AVT of around 50%, but the issue of photostability at higher temperatures might make them less well-suited for applications in warmer and high-humidity climates [36].

### 3.2. Super-Hydrophobic Surface Structuring for Stability

When considering large-scale commercial implementation of PVs, the three key factors to consider (or the “golden triangle”, as termed by Meng et al.) are low cost, a high PCE, and high stability, which translates to a long lifetime for the devices [37].

Both transparent and semi-transparent PVs are often required to operate outdoors, making their resistance to external factors, such as humidity, dust, and staining agents, an important characteristic for their successful implementation.

This is important, in general, for all types of photovoltaics: the presence of unwanted materials, such as ice, snow, plant matter, and others that make the surface of a PV device dirty, can lead to further reflection of light and loss in transparency, which, in turn, lowers the overall efficiency of the device [24,38].

A promising way of addressing this problem is by utilising biomimetic super-hydrophobic coatings with self-cleaning capabilities [24,39], especially since other technologies, such as autonomous cleaning sensors, are still at their infancy and have yet to reach commercial application.

A super-hydrophobic surface is commonly defined as one for which the contact angle of a drop of water on it is larger than 150° and its sliding angle (defined as the tilting angle of the surface at which the drop begins to slide on it) is lower than 5° [24], although a study conducted by Law suggests that 145° for the contact angle would be a better value for this definition [40]. When a drop of water is placed on such a surface, it rolls off very easily, generally carrying with it staining agents and cleaning the surface in the process, hence self-cleaning becomes a consequence of super-hydrophobicity.

It is worth pointing out, however, that super-hydrophobicity arises from a combination of chemical properties and high surface roughness, and that the latter is also a source of light-scattering: for this reason, super-hydrophobicity and anti-reflectance are competitive factors [41,42], so a good balance between the two must be achieved for photovoltaic applications.

There are several examples of super-hydrophobic surfaces in nature: from the leaves of several species of plants [43], with the most well-known being the lotus leaf [44,45], to insects’ wings [46,47,48,49] and compound eyes [50,51], including the already mentioned moth eyes [24,43,52].

**Figure 6:**
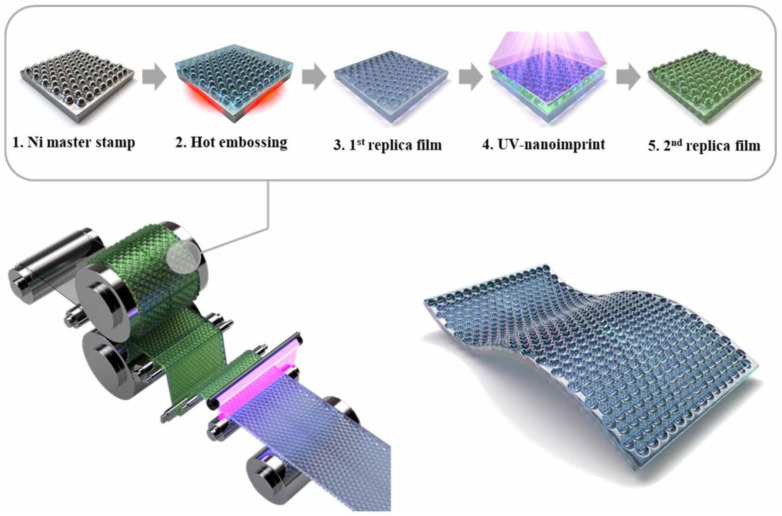
Schematic representation of the roll-to-roll printing process of the moth-eye film from Ju et al. Reprinted with permission from Ref. [53]. 2020, IOP Publishing.

An example of a biomimetic film with self-cleaning capabilities inspired by the latter can be found in the work of Ju et al. [53], in which a 170 nm high moth-eye pattern was fabricated on a 1.1 m wide polyethylene terephthalate (PET) film via roll-to-roll printing, making it suitable for large-scale photovoltaic applications (Figure 6). The resulting film was highly transparent, with an average transmittance in the visible of over 90%, and exhibited a contact angle of 140.3°; despite this value being lower than the one conventionally used to describe super-hydrophobic materials, the film still showed self-cleaning properties. The stability of these films was also tested in an oven at 60 °C and 80% humidity: after 168 h, the contact angle had only decreased to 139.8°, thereby demonstrating that the achieved super-hydrophobicity was stable, even in relatively challenging environments. Furthermore, the implementation of the moth-eye pattern reduced the visible reflectance by 3.2% and increased visible transmittance by 3.1%, on average, compared to the bare PET film, thus, improving performance for photovoltaic applications.

**Figure 7:**
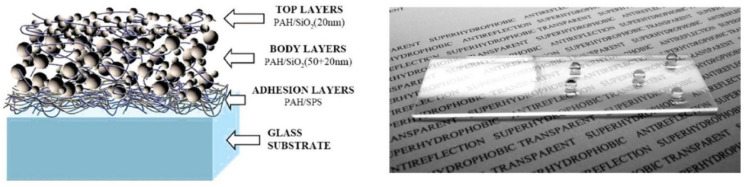
Schematic representation (**left**) of the transparent super-hydrophobic film and picture (**right**) of a glass slide coated with it showing both its transparency and super-hydrophobicity. Here, PAH stands for poly(allylaminehydrochloride) and SPS for Poly(sodium4-styrenesulfonate). Adapted with permission from Ref. [42]. 2007, American Chemical Society.

Another example of a transparent super-hydrophobic film can be found in the work of Bravo et al. [42], where the authors took inspiration from the lotus leaf. The super-hydrophobicity of this plant is a product of the presence of two levels of roughness (on the micrometric and on the nanometric scale); by applying this principle, a multi-layer film was fabricated utilising a mixture of 50 nm and 20 nm diameter SiO_2_ nanospheres directly on a glass surface, with the aid of polymeric layers to promote adherence to the surface and homogeneity of the resulting film (Figure 7). The film was then treated with silane to change its surface chemistry and confer super-hydrophobicity. The fabrication method only required submerging the substrate various times in different aqueous dispersions/solutions of nanospheres/polyelectrolytes, thus making it viable for large-scale applications. It is worth noting that different films were prepared with different numbers of nanosphere bilayers: the one achieving the highest AVT (around 90%) was the one with 20 bilayers. This film also showed anti-reflecting capabilities, with a lowest reflectance of around 0.22% at 562 nm and the highest value of 5.2% at 710 nm, compared with a reflectance of around 8% across all of the visible spectrum from the plain glass slide. If, however, an AVT value between 50–80% is acceptable for the intended purpose, a larger number of bilayers can be applied, which, in turn, confers excellent super-hydrophobic features. Further studies are, however, required regarding the stability over time of these films. Furthermore, both the cited examples would benefit from more extensive study regarding the permeability of the films to both oxygen and humidity, as these are detrimental factors to the performance of perovskite and organic photovoltaic cells. This kind of study would help in determining if they can also be used as encapsulants for such devices, or if they should be added on top of more conventional encapsulation techniques.

## 4. Future Research and Applications

The natural world can be a source of inspiration for promising applications to (s)TPVs. There are numerous additional natural systems that could potentially open new lines of research in this field, one of them being silk fibroins.

Silks produced by spiders or silkworms are the strongest fibres found in nature [54], with mechanical properties comparable to those of steel or Kevlar [55]. Furthermore, films made from silk fibroins extracted from the silkworm Bombyx Mori can exhibit an AVT of around 90% [56], are highly bio-compatible [57], and can be produced by using only aqueous solution processing, usually at near room temperature and ambient pressure, thereby making them more eco-friendly alternatives to more conventional materials, often non-biodegradable and derived from non-renewable sources [58,59].

**Figure 8:**
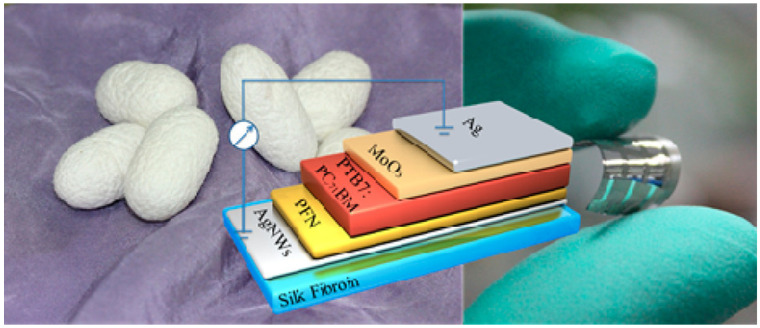
Bombyx Mori cocoons (**left**), schematic representation of the PV device (**centre**), and picture of the finished device (**right**). Here, PFN stands for poly[(9,9-bis(3′-(N,N-dimethylamino)propyl)-2,7-fluorene)-alt-2,7-(9,9-dioctylfluorene)] and PTB7 stands for poly [[4,8-bis[(2-ethylhexyl)oxy]benzo[1,2-b:4,5-b′]dithiophene-2,6-diyl][3-fluoro-2-[(2-ethylhexyl)carbonyl]thieno[3,4-b]thiophenediyl]]. Reprinted with permission from Ref. [60]. 2014, American Chemical Society.

Silk fibroin films have already been successfully used as a base for the fabrication of flexible, transparent substrates for organic light-emitting diodes (OLEDs) [59] and organic solar cells [60] by integrating a mesh of silver nanowires (AgNWs) into the film (Figure 8), and, thus, combining the function of electrode and mechanical support in the same device element. The resulting “conducting substrates” exhibit an AVT of ~80%, and a sheet resistance of ~11–12 Ω/sq, thus making them comparable with conventional ITO-coated polyethylene naphthalate (PEN)-based ones [59]. It was shown that such electrodes/substrates kept their good transport properties for at least up to 200 bending cycles [60]. High bio-compatibility, high AVT, resistance to mechanical stress, and the possibility of conferring good conductivity to the film make silk fibroin-based materials excellent candidates for the production of substrates for wearable devices, for which the direct contact with human skin makes bio-compatibility a fundamental requirement [60,61], together with high transparency, not detracting from the aesthetics of the final product. While on this topic, it is worth pointing out that silk fibroin-based materials can also be dyed, by introducing selected dyes directly into the diet of the silkworms: this allows the production of intrinsically coloured, or even fluorescent, silk, making the dyeing process much more environmentally sustainable and reducing the amount of chemical waste produced [62,63,64], as well as making silk fibroin-based materials suitable for applications where a specific colour is required.

Despite possessing these attractive characteristics, to the best of our knowledge very little research has been done on the implementation of silk fibroin-based materials for transparent photovoltaics, making it a good candidate for future (s)TPV developments.

On the topic of future developments, we would like to point out how the peculiar features of (s)TPVs make them uniquely suited for several future applications.

Alongside the improvement on materials and fabrication techniques for cheaper production and viable large-scale applications for BIPVs, BAPVs, and VIPVs, there are other fields in which bio-inspired (s)TPVs could find future novel applications, like the aforementioned wearable devices, smart glasses, or even smart contact lenses with added functionalities, like drug delivery and biosensing [65,66].

**Figure 9:**
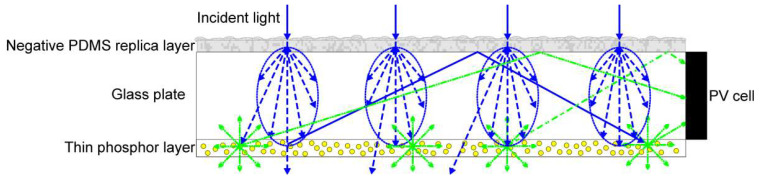
Schematic representation of the semi-transparent thin-film LSC implementing the negative PDMS replica layer of leaves’ microstructures. Reprinted with permission from Ref. [69]. 2022, license CC BY 4.0 (https://creativecommons.org/licenses/by/4.0/ (accessed on 20 October 2022)).

Further developments in the implementation of bio-inspired light-management strategies may also lead to improved performance of PV devices, as shown, for example, by Tsai et al. [67]. By implementing a moth-eye inspired embedded biomimetic nanostructure in their device design, the authors managed to improve the PCE of a hydrogenated amorphous silicon solar cell from 5.36% to 8.32%. This embedded biomimetic nanostructure had both anti-reflection and light-trapping properties, and, thus, led to improved performance of the device. Such a nanostructure, composed of an ordered array of nanospheres, closely resembles another structure from the natural world, namely that of the opal, which has also been shown to lead to improvements in the PCE of solar cells, thanks to its light-trapping properties [68]. Another type of PV device that may benefit from further developments in the implementation of bio-inspired light-management strategies is that of semi-transparent luminescent solar concentrators (LSCs), as shown, for instance, in a recent work by Chen et al. [69]. Here, a polydimethylsiloxane (PDMS) negative replica layer of leaves’ microstructures conferred improved light-scattering properties to the device (i.e., the “haze” or the portion of light diffusely scattered through a transparent surface with respect to the total light transmitted through such interface). This is beneficial as it leads to a greater amount of light being scattered into the LSC and into the thin luminescent phosphor layer placed on its bottom (Figure 9).

Overall, the implementation of biologic or bio-inspired materials and biomimetic strategies may indeed prove to be beneficial for future developments, both in already established technologies and in new emerging fields.

## 5. Conclusions

In this mini-review we have shown how a biomimetic approach to (s)TPVs can offer avenues to explore in the attempt to address the various key challenges in this field, by providing effective ways of improving the performance of devices, without detracting from their aesthetics, and making them more stable in non-controlled environments. New biomaterials can open new paradigms for research and applications in the field, such as fully bio-compatible (s)TPVs, while also providing a much greener approach to their fabrication.

## Data Availability

Not applicable.
